# Developing a methodology for three-dimensional correlation of PET–CT images and whole-mount histopathology in non-small-cell lung cancer

**DOI:** 10.3747/co.v15i5.349

**Published:** 2008-10

**Authors:** M. Dahele, D. Hwang, C. Peressotti, L. Sun, M. Kusano, S. Okhai, G. Darling, M. Yaffe, C. Caldwell, K. Mah, J. Hornby, L. Ehrlich, S. Raphael, M. Tsao, A. Behzadi, C. Weigensberg, Y.C. Ung

**Affiliations:** * Radiation Medicine Program, Princess Margaret Hospital, University Health Network, Toronto, ON; † Department of Radiation Oncology (Dahele, Ung), Department of Pathology (Hwang, Mah, Raphael, Tsao), Division of Thoracic Surgery (Darling), Department of Medical Imaging (Ehrlich, Yaffe, Caldwell), and Department of Medical Biophysics (Yaffe, Caldwell), University of Toronto, Toronto, ON; ‡ Department of Pathology (Hwang, Tsao) and Division of Thoracic Surgery (Darling, Hornby), Toronto General Hospital, University Health Network, Toronto, ON; § Department of Imaging Research, Sunnybrook Research Institute (Peressotti, Sun, Kusano, Okhai, Yaffe, Caldwell, Mah); Department of Medical Physics (Mah); Department of Medical Imaging (Ehrlich); and Department of Pathology (Raphael), Sunnybrook Health Sciences Centre, Toronto, ON; || Department of Surgery (Behzadi) and Department of Pathology (Weigensberg), The Scarborough Hospital, Toronto, ON; # Department of Radiation Oncology, Odette Cancer Centre, Toronto, ON

**Keywords:** Radiology pathology correlation, positron emission tomography, non-small-cell lung cancer

## Abstract

**Background:**

Understanding the three-dimensional (3D) volumetric relationship between imaging and functional or histopathologic heterogeneity of tumours is a key concept in the development of image-guided radiotherapy. Our aim was to develop a methodologic framework to enable the reconstruction of resected lung specimens containing non-small-cell lung cancer (nsclc), to register the result in 3D with diagnostic imaging, and to import the reconstruction into a radiation treatment planning system.

**Methods and Results:**

We recruited 12 patients for an investigation of radiology–pathology correlation (rpc) in nsclc. Before resection, imaging by positron emission tomography (pet) or computed tomography (ct) was obtained. Resected specimens were formalin-fixed for 1–24 hours before sectioning at 3-mm to 10-mm intervals. To try to retain the original shape, we embedded the specimens in agar before sectioning. Consecutive sections were laid out for photography and manually adjusted to maintain shape. Following embedding, the tissue blocks underwent whole-mount sectioning (4-μm sections) and staining with hematoxylin and eosin. Large histopathology slides were used to whole-mount entire sections for digitization. The correct sequence was maintained to assist in subsequent reconstruction.

Using Photoshop (Adobe Systems Incorporated, San Jose, CA, U.S.A.), contours were placed on the photographic images to represent the external borders of the section and the extent of macroscopic disease. Sections were stacked in sequence and manually oriented in Photoshop. The macroscopic tumour contours were then transferred to MATLAB (The Mathworks, Natick, MA, U.S.A.) and stacked, producing 3D surface renderings of the resected specimen and embedded gross tumour. To evaluate the microscopic extent of disease, customized “tile-based” and commercial confocal panoramic laser scanning (TISSUEscope: Biomedical Photometrics, Waterloo, ON) systems were used to generate digital images of whole-mount histopathology sections. Using the digital whole-mount images and imaging software, we contoured the gross and microscopic extent of disease.

Two methods of registering pathology and imaging were used. First, selected pet and ct images were transferred into Photoshop, where they were contoured, stacked, and reconstructed. After importing the pathology and the imaging contours to MATLAB, the contours were reconstructed, manually rotated, and rigidly registered. In the second method, MATLAB tumour renderings were exported to a software platform for manual registration with the original pet and ct images in multiple planes. Data from this software platform were then exported to the Pinnacle radiation treatment planning system in dicom (Digital Imaging and Communications in Medicine) format.

**Conclusions:**

There is no one definitive method for 3D volumetric rpc in nsclc. An innovative approach to the 3D reconstruction of resected nsclc specimens incorporates agar embedding of the specimen and whole-mount digital histopathology. The reconstructions can be rigidly and manually registered to imaging modalities such as ct and pet and exported to a radiation treatment planning system.

## 1. INTRODUCTION

Lung cancer is the leading cause of cancer death in Canada, and it is associated with one of the lowest survival rates of all cancers 1. Radiotherapy is frequently used in the treatment of lung cancer 2. Radiotherapy is a paradigm for image-guided therapies that include surgical and interventional radiology techniques. Image-guided therapies have in common a need to understand the relationship between what is visualized on imaging [for example, computed tomography (ct)] and the “true” pathologic extent of gross and microscopic disease. This need was the motivation for the study described here.

In the case of radiotherapy, the accurate delineation of the gross tumour volume (gtv) is a critical step in treatment planning. However, there are currently several weak links in this process. The significant inter-observer variation that occurs in ct based gtv 3 is only partially reduced by the integration of ^18^F fluorodeoxyglucose (fdg) positron emission tomography (PET) imaging 4. Also, it is unclear how large a margin should be added to the gtv to account for microscopic tumour extension to generate the clinical target volume. Therefore, in practice, a uniform 5-mm to 10-mm margin is often added around the gtv, on the assumption that the ct-based gtv is inadequate. Such shortcomings provide a rationale for studies to investigate whether what is seen on ct or pet imaging accurately reflects the underlying pathologic extent of disease in nsclc and how best to view and segment ct and pet images to reflect that pathology.

**FIGURE 1:**
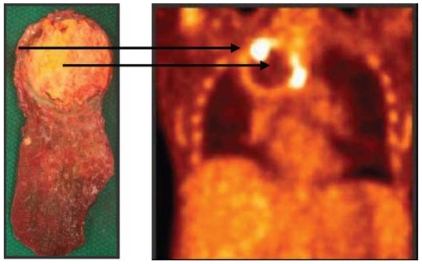
Complete pathologic response following induction chemotherapy and chemoradiation. A fibrotic rim surrounds a necrotic core. The preoperative ^18^F fluorodeoxyglucose pet scan demonstrates corresponding areas of rim enhancement and a photopenic centre (arrows).

Radiology–pathology correlation (rpc) is one investigational approach to this problem (Figure 1). Several authors have described some form of rpc in NSCLC 5–10, using techniques that vary in complexity. Giraud *et al.* 5 used a two-dimensional methodology. These authors examined 354 pathology slides from 70 lobectomy or pneumonectomy specimens that had been insufflated with 10% formalin. They identified the macroscopic extent of disease by naked eye and found that their observations correlated well (*p* < 0.001) with tumour dimension on ct scans at the “optimal window/level setting” (which was undefined). They further determined that margins of 8 mm for adenocarcinomas and 6 mm for squamous carcinomas were needed to account for microscopic extension with 95% probability. They recognized that their calculations were affected by the degree of insufflation of the specimen. The recently reported pilot study by Stroom *et al.* 10 used more advanced methodology to begin to account for *ex vivo* changes in tissue dimensions and geometry.

Such studies make it apparent that robust rpc in the lung is a difficult undertaking. However, few papers speak directly to the challenges that are encountered in this work. In this qualitative report of our initial experience with developing a three-dimensional (3D) methodology for pet or ct rpc in nsclc, we discuss some of these challenges, report our methodology, and highlight future areas of research.

Our aim was to develop a methodology that would achieve these ends:

- Fix the resected specimen in a shape and size reflective of the *in vivo* state
- Maintain the spatial orientation of the specimen and sections throughout the process
- Enable the assessment of macroscopic and microscopic disease
- Permit a 3D reconstruction of resected specimens
- Allow for specimen contours to be registered with pet or ct images and to be imported into a commercial radiation treatment planning system

## 2. METHODS AND RESULTS

With institutional research ethics board approval, we recruited 12 patients to a study designed to investigate rpc in nsclc (Table I). All patients were scheduled to undergo surgical resection of nsclc, with or without induction chemoradiation. Because the optimum means of fixing pathology tissue for rpc has yet to be determined and was to be investigated, it was stipulated that whatever tissue would ordinarily be taken for routine clinical pathology reporting would continue to be acquired so as not to compromise patient management. This necessary requirement, combined with the small size of most of the tumours (7 were 3 cm or smaller), reduced the amount of tissue that was available for study purposes.

Throughout the course of the study, each specimen was processed so as to try to improve on the preceding effort. The resulting methodology is described.

### 2.1 Preoperative Imaging and Handling of the Resected Specimen

All patients underwent combined fdg pet or ct imaging before tumour resection. These operative techniques were used:

- Wedge resection and segmentectomy (*n* = 3)
- Lobectomy (*n* = 8)
- Pneumonectomy (*n* = 1)

One specimen incorporated a chest wall resection and was unsuitable for further analysis. The remaining specimens (11 of 12) were insufflated with 10% formalin by endobronchial or transparietal injection, or both. Injection ceased when the specimen was saturated and formalin was being exuded across the pleura. Where necessary, surfaces were inked to aid orientation. At that point, the specimen was photographed with an accompanying centimetre scale. It was then immersed in formalin for a time that varied from 1 to 24 hours for initial fixation.

### 2.2 Sectioning and Processing the Specimen

After initial fixation, the specimens were sectioned at intervals of approximately 3–10 mm. The choice of location and the direction of the first cut was made on clinical grounds, and all subsequent sections were parallel to the first. A very sharp blade is required to minimize shear and tearing when cutting through different tissues simultaneously (for example, lung parenchyma and cartilage). Various methods of sectioning were tried, including freehand knife, customized sectioning box (metal box designed to contain the specimen during cutting), and electric rotary cutter. Freehand cutting was the least reliable, and in general, the combination of the electric rotary cutter and agar embedding (discussed next) provided the most consistent sections.

The duration of initial fixation made little difference to the rigidity of the specimen. Even prolonged fixation was insufficient to ensure alignment of the successive sections. In addition, when the inflated gross specimen was cut, the retained formalin ran out, causing specimens to deflate to a greater or lesser extent, contributing to the variability of sections and reducing the likelihood that radiology and pathology would correlate without some form of image registration and deformation. These particular techniques were not used in this study, which focused on the physical presentation of the specimen.

**FIGURE 2:**
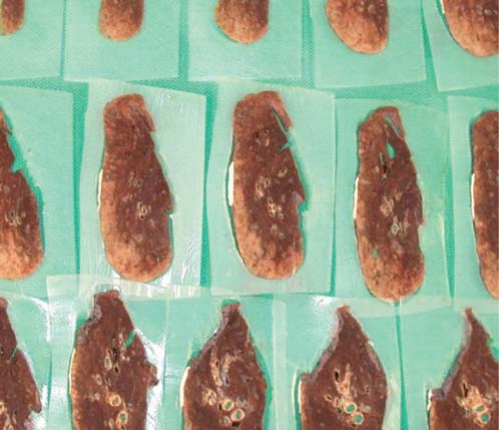
The utility of embedding specimens in agar after initial formalin fixation was studied. In selected cases, it helped to maintain the conformation of sections.

To try to retain shape, some of the specimens were embedded in agar before sectioning. This technique surrounds the specimen with a 3.5% agar mixture, initially at 55°C before cooling 11. Before the agar was used, it was first applied to regions of lung tissue remote from the tumour. An experienced lung pathologist then examined tissue sections and determined that no significant microscopic tissue damage had occurred as a result of the temperature used in the process. However, despite preparing the pleural surface with alcohol, the lung did not reliably stick to the agar. As a result, in cases in which little collapse of the specimen occurred on sectioning, the agar helped to maintain conformation of the sections (Figure 2). But where the specimen collapsed significantly or where shearing on cutting occurred, the agar made less difference.

The agar method has benefits, but additional work is needed to refine the physical preparation of lung specimens in rpc.

**FIGURE 3:**
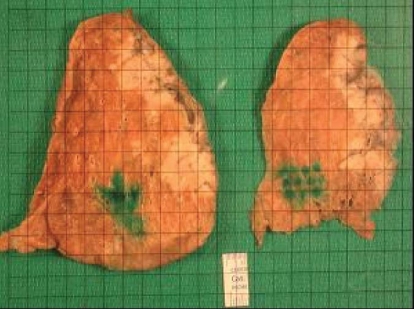
The resected specimen is sectioned after insufflation with 10% formalin and is photographed with an overlying grid (pale tumour is seen on the right of each section).

The naked-eye extent of macroscopic disease was measured at sectioning by an experienced lung pathologist. Consecutive sections were laid out for photography and were manually adjusted to maintain shape. Excess surface formalin was removed, and the sections were digitally photographed with an accompanying scale or centimetre grid (Figure 3). Where possible, each specimen was aligned with the central axis of the camera at a fixed distance from the lens and with the scale at the same height as the uppermost surface of the section.

**FIGURE 4:**
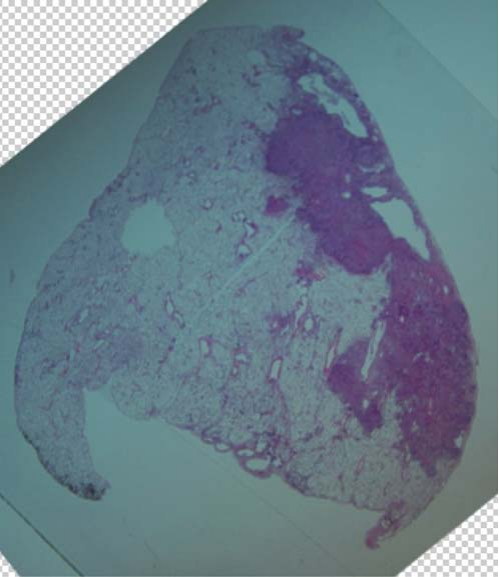
After additional processing, whole-mount sections 4 μm in thickness are cut and stained with hematoxylin and eosin.

After the necessary removal of samples for clinical reporting, the remaining sections from a total of 4 specimens underwent tissue processing and paraffin embedding (this step was governed by the availability and suitability of remaining tissue, which were influenced by such factors as tumour size and location). Following embedding, the tissue blocks underwent whole-mount sectioning (4 μm sections) and staining with hematoxylin and eosin. Large histopathology slides (for example, 5 × 3 inches) were used to whole-mount entire sections in preparation for digitization (Figure 4). The correct sequence was maintained, and the location of any slices that had been removed was noted to assist in the subsequent volumetric reconstruction.

### 2.3 Identifying and Reconstructing Gross and Microscopic Disease

**FIGURE 5:**
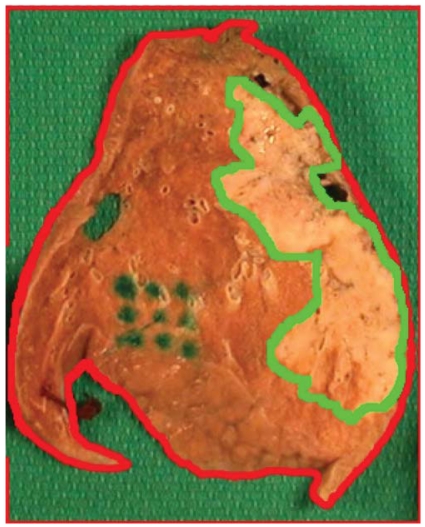
The gross section is photographed, and the lung parenchyma, visible tumour, and any intrinsic fiducial landmarks are contoured using Photoshop (Adobe Systems Incorporated, San Jose, CA, U.S.A.).

Using Photoshop (Adobe Systems Incorporated, San Jose, CA, U.S.A.), contours were placed on the photographic images to represent the external border of the section, and the extent of macroscopic disease (Figure 5). These sections were stacked in sequence and manually oriented in Photoshop using natural contours and intrinsic fiducial landmarks (for example, bronchi). To maintain the clinical integrity of the specimen, implanted fiducial markers were not used in the present study, although it is likely that such markers would have improved the registration of sections. Such markers would also have been of use in evaluating image deformation.

**FIGURE 6:**
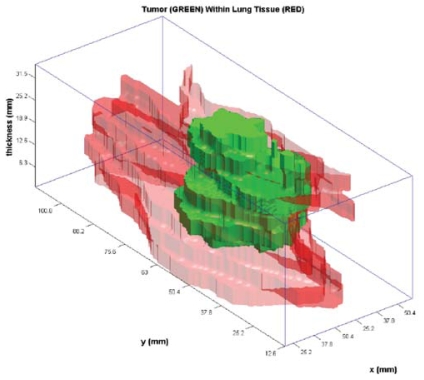
The contours from digital photographs are transferred into MATLAB (The Mathworks, Natick, MA, U.S.A.) and expanded to generate a three-dimensional rendering of the resected specimen. A contiguous outer surface relies on conformation of the sections being retained between sectioning and laying out the specimen. No extrapolation was used between sections. Green = reconstructed tumour; red and pink = surrounding lung parenchyma.

The macroscopic tumour contours were then transferred to MATLAB, where they were stacked at an appropriate distance apart (compensating for missing slices where necessary). The stacking resulted in 3D surface renderings of the resected specimen and embedded gross tumour (Figure 6).

**FIGURE 7:**
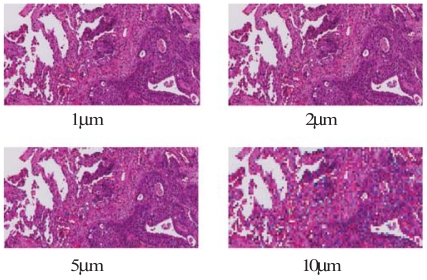
Digitised histopathology is generated from the whole-mount sections. The resolution can be varied. We determined that 2 μm was adequate for identifying microscopic disease.

To evaluate the microscopic extent of disease, customized “tile-based” 12 and commercial confocal panoramic laser scanning [TISSUEscope (Biomedical Photometrics, Waterloo, ON)] systems were used to generate digital images of complete whole-mount histopathology sections (Figure 7). We were able to generate at least 1 good quality whole-mount section in 4 patients. Digital whole-mount pathology images of varying spatial resolution—1, 2, 5, and 10 μm—were evaluated by experienced lung pathologists to determine the resolution adequate for assessing microscopic disease. The expert consensus was to use a resolution of 2 μm. Using the digital whole-mount images and imaging software, a lung pathologist contoured both the gross and the microscopic extent of disease. Had sufficient sequential slides been available to be processed in this fashion, the slides could then have been reconstructed to provide a 3D histopathologic representation of the tumour.

### 2.4 Registering Pathology and Imaging Data and Exporting Information to the Radiation Treatment Planning System

**FIGURE 8:**
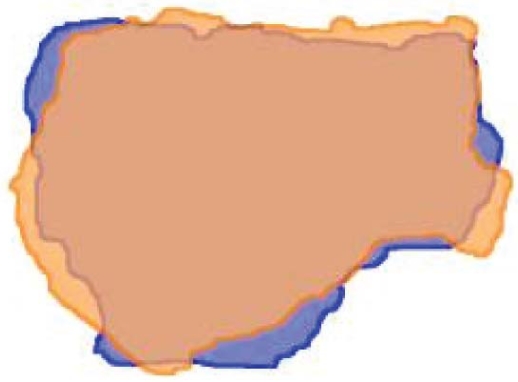
Rigid (manual) registration in three dimensions of the maximum projections on computed tomography (ct, orange) and gross tumour as identified on digital photograph (blue).

**FIGURE 9:**
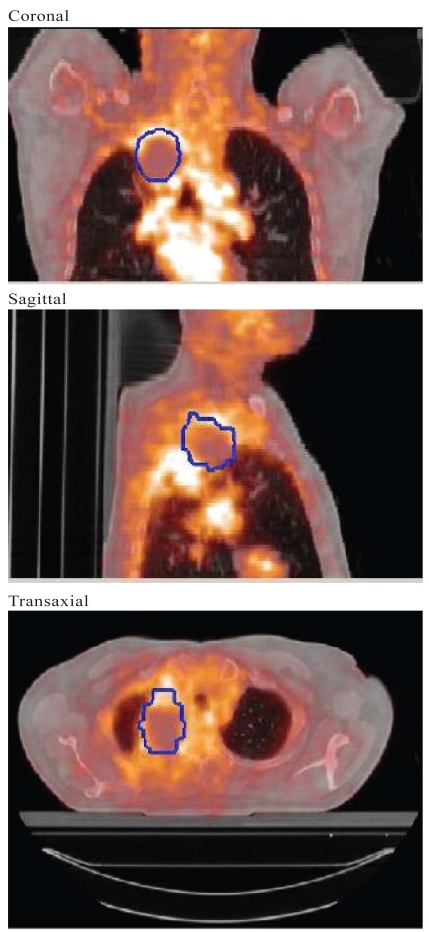
Manual registration in three planes between tumour (blue contour) reconstructed using digital photographs [Photoshop (Adobe Systems Incorporated, San Jose, CA, U.S.A.) and MATLAB (The Mathworks, Natick, MA, U.S.A.)] and a fused pet–ct image.

Two methods of registering pathology and imaging were used. In the first, selected pet or ct images were transferred into Photoshop (ensuring correct scaling of the images). These images were then contoured, stacked, and reconstructed as described earlier. A range of window and level settings and segmentation approaches can be used to generate the pet or ct tumour boundaries. After the pathology and imaging contours are imported to MATLAB, they can be reconstructed, manually rotated, and rigidly registered (Figure 8). In the second method, MATLAB tumour renderings were exported to a software platform that permitted manual registration with the original pet or ct images in the axial, coronal, and sagittal planes (Figure 9).

Either of the foregoing registration methods allows for pathology-based and imaging-based volumes (at various window and level and segmentation thresholds) to be measured. Differences in the volumes give some idea of relative changes in the tissues between the *in vivo* and *ex vivo* states.

Data from this software platform were then exported to the Pinnacle^3^ (Philips Medical Systems, Cleveland, OH, U.S.A.) radiation treatment planning system in dicom (Digital Imaging and Communications in Medicine) format. This is one method by which pathology information can be integrated into the study of radiation treatment planning.

### 2.5 Image Registration and Deformation and Overall Accuracy of the Method

**FIGURE 10:**
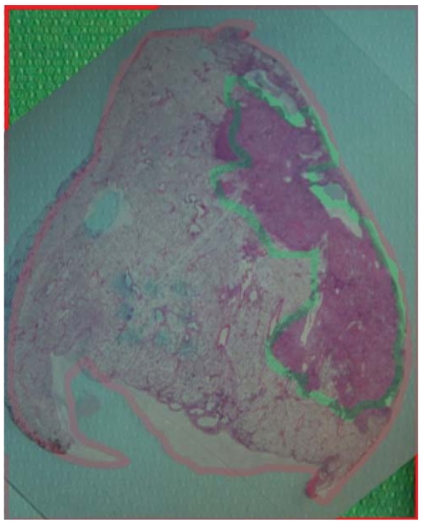
Manual superposition of whole-mount histopathology and corresponding digital photograph. Red and green contours on the photograph represent specimen edge and naked-eye gross tumour respectively. This superposition illustrates that some mismatch remains, which could be the result of changes in conformation and specimen dimension during sectioning and processing.

A change occurs in the 3D conformation of tissues between the *in vivo* and *ex vivo* states. Reasons for this change include the physical properties of lung tissue, with its tendency to collapse, which can lead to the systematic propagation of conformational changes throughout the study process and specimen processing (Figure 10). It is difficult to compensate for these changes, and physical efforts such as those discussed here are likely to benefit from the addition of sophisticated image registration and deformation technologies.

In considering the overall accuracy of any method for rpc in nsclc, the difference between what is often added empirically for microscopic extension in radiation treatment planning and what was actually demonstrated by Giraud *et al.* is noted typically to be less than 5 mm. Therefore, to generate clinically useful information, an rpc methodology arguably needs to be accurate to 1–2 mm. Our experience, and the reported literature, indicates many possible sources of (cumulative) measurement error that have not yet been accurately quantified.

## 3. DISCUSSION AND CONCLUSIONS

Radiology–pathology correlation in lung is challenging. It requires a range of expertise and logistics coordination and must not compromise clinical reporting. No single definitive method has been established for 3D volumetric rpc in nsclc. The present report describes an innovative approach to the 3D reconstruction of resected nsclc specimens. The methodology incorporates several distinguishing features, including agar embedding of the specimen and whole-mount digital histopathology. As described, the reconstructions can be rigidly and manually registered (with good approximation) to imaging modalities such as ct and pet and then exported to a radiation treatment planning system. The methodologic principles are expected to be transferable to other tumour sites 13 and to support a range of investigative possibilities 14. With the addition of more precise techniques for image registration and deformation 15, the overall accuracy of the method should be improved, enabling the acquisition of quantitative information. To develop robust rpc techniques, factors such as the timing between imaging and surgery, and the use of respiratory-correlated pet and ct imaging will become relevant. In conclusion, therefore, we have developed a framework for rpc (Appendix a) and identified discrete areas that would benefit from further research and refinement.

## 4. PROJECT SUPPORT

The present work was supported by a grant from the Ontario Cancer Research Network.

## Figures and Tables

**TABLE I:** Characteristics of the study population

Patient	Operation	Histology	Maximum gross tumour dimension
1	rll	Squamous	10 cm
2	rul + chest wall resection a	Adenocarcinoma	8 cm
3	rul	Adenocarcinoma	3.2 cm
4	Segmentectomy	Adenocarcinoma	3 cm
5	Segmentectomy	Squamous	2.9 cm
6	rul	bac/adenocarcinoma	2.5 cm
7	lul	Adenocarcinoma	2.1 cm
8	rul	Adenocarcinoma	2 cm
9	Wedge resection	Squamous	1.6 cm
10	lll	Adenocarcinoma	1.2 cm
11	rul	nsc carcinoma	No residual tumour b (Figure 1)
12	Pneumonectomy	Large-cell carcinoma	No residual tumour b

a Not suitable for study because of attached chest wall.

b Complete pathologic response following chemoradiation.

rll = right lower lobectomy; rul = right upper lobectomy; bac = bronchoalveolar carcinoma; lul = left upper lobectomy; lll = left lower lobectomy; nsc = non-small-cell.

## APPENDIX A

Development of a framework for three-dimensional radiology–pathology correlation (rpc)

1. Preoperative positron emission tomography (pet) or computed tomography (ct) imaging
2. Surgical resection
3. Specimen insufflation with formalin
4. Specimen embedding in agar
5. Specimen sectioning and gross tumour measurement
6. Digital photograph of sections
7. Digital photograph-based tumour reconstruction and rpc:
  - Transfer digital images of sections to Photoshop (Adobe Systems Incorporated, San Jose, CA, U.S.A.).
  - Contour gross tumour and specimen edges, as well as any internal fiducial markers (for example, bronchi).
  - Perform rigid manual registration and stacking of the contours in Photoshop.
  - Export the stacked contours to MATLAB (The Mathworks, Natick, MA, U.S.A.), and reconstruct the tumour and lung specimen.
  - Repeat the foregoing four steps with sequential cross-sectional images of the tumour.
  - Manually register the reconstructed pathology and radiology in MATLAB.
  - Alternatively, export the pathology reconstruction from MATLAB to another platform that allows for registration of reconstructed pathology with diagnostic pet or ct images in three dimensions, then export data from that platform to the radiation treatment planning system.
8. Histopathology-based tumour reconstruction:
  - Process pathology sections, stain with hematoxylin and eosin, and generate 4 μm whole-mount sections.
  - Digitize pathology using tiling-based system or confocal laser scanning.
  - Import digital images into a software platform for contouring of gross and microscopic tumour at 2 μm resolution.
  - Reconstruct tumour and specimen using the contours created in the immediately preceding step.
  - Register with radiology as described for photograph-based reconstruction.
